# Shoulder strap fixation of LUCAS-2 to facilitate continuous CPR during non-supine (stair) stretcher transport of OHCAs patients

**DOI:** 10.1038/s41598-021-89291-4

**Published:** 2021-05-10

**Authors:** Chen-Bin Chen, Kuan-Fu Chen, Cheng-Yu Chien, Chan-Wei Kuo, Zhong Ning Leonard Goh, Chen-Ken Seak, Joanna Chen-Yeen Seak, Chen-June Seak, For the SPOT Consortium, Johan Seak, Chia Hsun Chang, Li-Heng Tsai, Chip-Jin Ng, Hsien-Yi Chen, Yu-Shao Chou, Tzu-Heng Cheng, Chia-Hau Chang, Chien-Lin Chen, Chiao-Hsuan Hsieh

**Affiliations:** 1 Department of Emergency Medicine, Lin-Kou Medical Center, Chang Gung Memorial Hospital, Taoyuan, Taiwan; 2 College of Medicine, Chang Gung University, Taoyuan, Taiwan; 3 Department of Emergency Medicine, New Taipei Municipal Tucheng Hospital, New Taipei City, Taiwan; 4 Department of Emergency Medicine, Chang Gung Memorial Hospital, Keelung, Taiwan; 5 Clinical Informatics and Medical Statistics Research Center, Chang Gung University, Taoyuan, Taiwan; 6 Community Medicine Research Center, Chang Gung Memorial Hospital, Keelung, Taiwan; 7 Department of Emergency Medicine, Ton-Yen General Hospital, Zhubei, Taiwan; 8 Sarawak General Hospital, Kuching, Sarawak Malaysia

**Keywords:** Diseases, Medical research, Signs and symptoms

## Abstract

Early recognition and rapid initiation of high-quality cardiopulmonary resuscitation (CPR) are key to maximising chances of achieving successful return of spontaneous circulation in patients with out-of-hospital cardiac arrests (OHCAs), as well as improving patient outcomes both inside and outside hospital. Mechanical chest compression devices such as the LUCAS-2 have been developed to assist rescuers in providing consistent, high-quality compressions, even during transportation. However, providing uninterrupted and effective compressions with LUCAS-2 during transportation down stairwells and in tight spaces in a non-supine position is relatively impossible. In this study, we proposed adaptations to the LUCAS-2 to allow its use during transportation down stairwells and examined its effectiveness in providing high-quality CPR to simulated OHCA patients. 20 volunteer emergency medical technicians were randomised into 10 pairs, each undergoing 2 simulation runs per experimental arm (LUCAS-2 versus control) with a loaded Resusci Anne First Aid full body manikin weighing 60 kg. Quality of CPR compressions performed was measured using the CPRmeter placed on the sternum of the manikin. The respective times taken for each phase of the simulation protocol were recorded. Fisher’s exact tests were used to analyse categorical variables and median test to analyse continuous variables. The LUCAS-2 group required a longer time (~ 35 s) to prepare the patient prior to transport (p < 0.0001) and arrive at the ambulance (p < 0.0001) compared to the control group. The CPR quality in terms of depth and rate for the overall resuscitation period did not differ significantly between the LUCAS-2 group and control group, though there was a reduction in both parameters when evaluating the device’s automated compressions during transport. Nevertheless, the application of the LUCAS-2 device yielded a significantly higher chest compression fraction of 0.76 (p < 0.0001). Our novel adaptations to the LUCAS-2 device allow for uninterrupted compressions in patients being transported down stairwells, thus yielding better chest compression fractions for the overall resuscitation period. Whether potentially improved post-OHCA survival rates may be achieved requires confirmation in a real-world scenario study.

## Introduction

Survival rates following out-of-hospital cardiac arrests (OHCAs) have improved significantly over the years to the current 8–12%^1,2^. Nevertheless, there is still room for improvement in patient management at all points in the clinical course of an OHCA, especially early recognition of cardiac arrest and rapid initiation of high-quality cardiopulmonary resuscitation (CPR)^3^. These aspects help maximise chances of achieving successful return of spontaneous circulation (ROSC), as well as improving patient outcomes both inside and outside the hospital^4,5^.

High-quality CPR comprises chest compressions of adequate rate and depth without interruption, coupled with adequate, non-excessive ventilation. The ability of a medical personnel to deliver high-quality CPR depends on personal skill and endurance; fatigue during long periods of continuous resuscitation can contribute to declining quality^6^. In fact, emergency medical technicians (EMTs) have been found to typically perform effective chest compressions only 50% of the time during resuscitation^7^. Manual CPR is also hampered and often impossible during transportation of the patient. Besides chest compressions, effective and adequate oxygenation of the brain is important in maximising neurological outcomes^8–13^.

To address these issues, mechanical chest compression devices like the LUCAS-2 (Jolife AB; Lund, Sweden) have been developed to assist rescuers in providing consistent, high-quality compressions, even during transportation^14–18^. Outsourcing of chest compressions to a mechanical device also has the added benefit of allowing rescuers to focus on securing the airway via placement of endotracheal tubes or laryngeal mask airways, thus ensuring proper oxygenation while automated chest compressions are ongoing. These devices however can only be applied to patients in a supine position. This limitation renders patients who have to be transported down stairwells and across tight spaces unable to benefit from such a device. Consequently, it represents a huge barrier to the further improvement of CPR quality in countries such as Taiwan where 83.7% of all residency buildings lack elevators^19^, and exacerbated by the significant delay faced by EMTs in accessing patients, especially those located three or more floors above ground^20^.

In order to allow the application of LUCAS-2 in these OHCA patients to minimize interruption and maintain high quality CPR during transportation down stairwells, we devised simple adaptations in the form of shoulder straps. This study aims to examine the effectiveness of our new adaptations to the chest compression device in providing high-quality CPR to simulated OHCA patients.

## Materials and methods

### Study setting

This non-randomised manikin simulation trial was conducted between December 3, 2017 and March 18, 2018. A simulation protocol was set up in accordance with the 2015 American Heart Association (AHA) Basic Life Support (BLS) guidelines^21^. This study was approved by the Chang Gung Memorial Hospital Institutional Review Board (IRB No.20170044B0).

### Study materials

A loaded Resusci Anne First Aid full body manikin (Laerdal; Stavanger, Norway) was used to simulate a 60-kg OHCA patient, with a pair of volunteer EMTs forming each medical response team. The LUCAS-2 chest compression system was used for mechanical automated chest compressions. This system is a mechanical CPR device with an integrated piston and suction cup designed to deliver compressions according to resuscitation guidelines, and has been experimentally shown to improve organ perfusion pressures, enhance cerebral blood flow, and increase end-tidal CO_2_ tension compared to manual CPR^22,23^.

**Figure 1:**
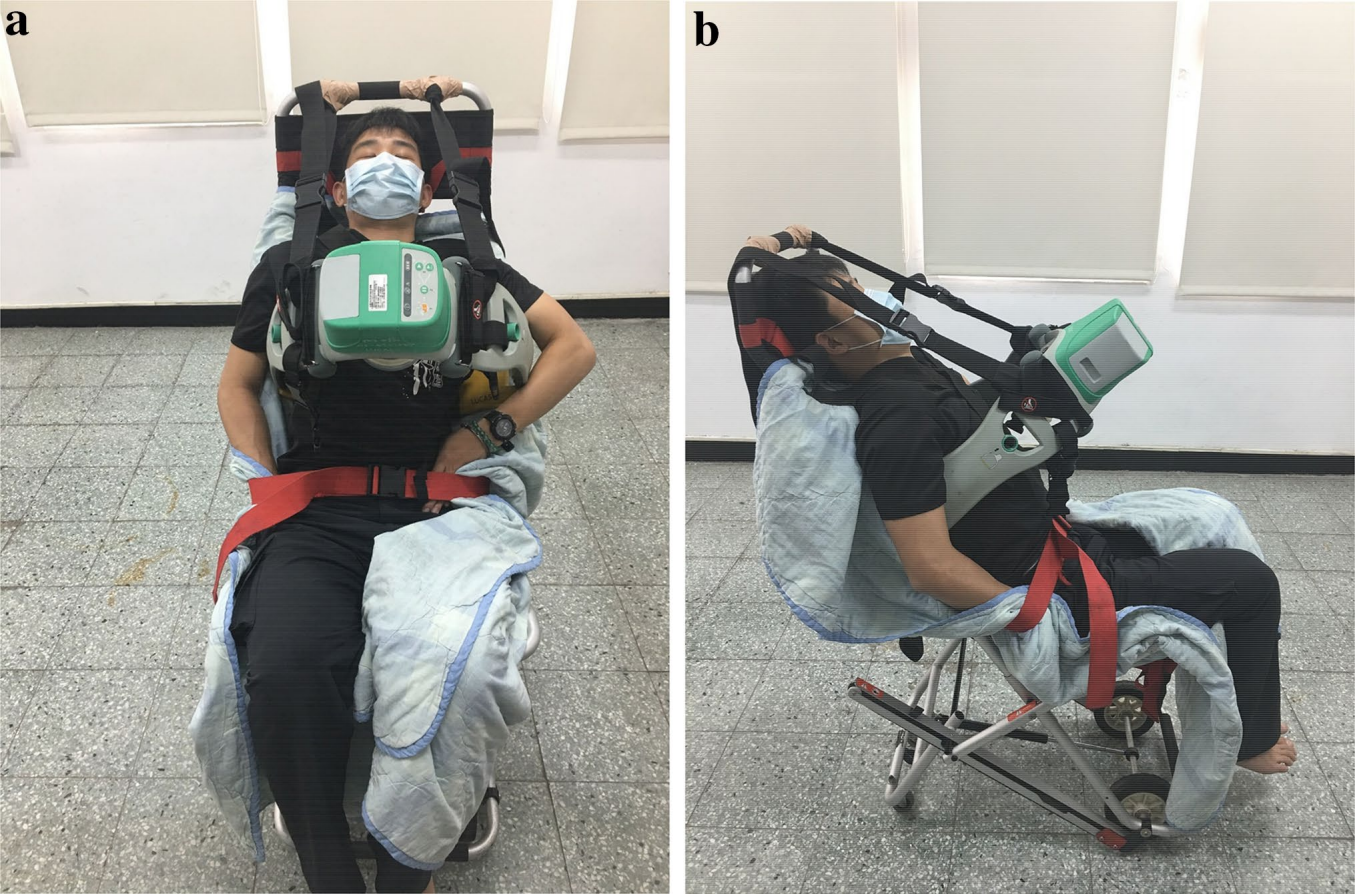
Adaptations of LUCAS-2 mechanical chest compression device enabling its operation in upright position during transportation. (**a**) Anterior view of simulated patient. (**b**) Lateral view of simulated patient.

To enable operation of LUCAS-2 in a non-supine position while the patients are transported on the stair stretcher, we fixed adjustable attachment straps to various points of the device. The straps along both sides of the upper edge of the backboard, to be secured to the lower edge on the opposite side (Fig. 1a,b), served to bind the backboard to the OHCA patient by going from the ipsilateral shoulder to the contralateral axilla. Two more sets of straps on the sides of the LUCAS-2 device helped to improve the stability of the entire contraption and prevent slippage. Quality of CPR compressions performed was measured using the CPRmeter (Laerdal; Stavanger, Norway) placed on the sternum of the manikin prior to the start of each simulation run.

### Simulation protocol

We set up simulated scenarios in accordance with the 2015 AHA BLS guidelines^21^ at the scene and during transportation. Each OHCA simulated patient, positioned on the third floor of a five-storied building without an elevator, was tended to by a team of two EMTs. The scenario required the EMTs to identify the patient’s cardiac arrest, immediately initiate manual chest compressions, and attach the training automated external defibrillator (AED) for initial rhythm analysis and shock delivery. As per standard practice, 2 min of CPR and laryngeal mask placement was then performed, before further actions were taken.

Subsequent management protocol differed according to group. The experimental group was instructed to install the adapted LUCAS-2 device on the manikin before transporting it down the stairs in a stair stretcher, while the control group was tasked to strap the manikin directly to the stair stretcher and bring it to the ground floor. Installation of the LUCAS-2 device was done by aligning the piston directly with the accelerometer of the CPRmeter manually, with the device’s continuous compression mode selected post-installation.

Bag valve mask ventilation was carried out as per usual CPR protocols during patient transport down the stairwell for both groups. The scenario was considered completed upon the EMT team’s arrival at the ambulance on the ground floor. Post-simulation, the positions of the LUCAS-2 device piston in relation to the CPRmeter accelerometer were inspected to check for slippage. A sample video of the trial on a simulated patient is available as Supplementary Video 1 (https://www.dropbox.com/s/kdq0yk4cenaopsx/%E4%B8%8A%E5%AD%97%E5%B9%95.wmv?dl=0). Informed consent to publish was obtained from all participants in the video.

### Participants

20 EMTs with a minimum of EMT-II certification (Table 1) were recruited from the city fire brigade and placed in teams of two. All EMTs had more than a year’s working experience and had participated in more than five OHCA rescue missions per year. Basic demographic details were recorded, such as age, sex, EMT level, and years of working experience.

**Table 1:** EMT certification requirements.

EMT level	Pre-requisites	Duration of training (h)	Skills
EMT-I	Not applicable	40	Basic Life Support techniques, including: oropharyngeal and nasopharyngeal airway access, provision of oxygen
EMT-II	EMT-I license	280	Laryngeal mask insertion, intravenous infusion, administration of medication
EMT-P	EMT-II license 4 years of experience OR college degree	1280	Advanced Life Support procedures

*EMTs* emergency medical technicians.

All participants were trained on placement of the adapted LUCAS-2 device on the manikin with immediate feedback provided. Each EMT was asked to demonstrate the technique thrice before the actual simulation test to ensure that training was effective. After the device placement training session, participants were provided 10 min to have hands-on practice on the manikin with the CPR meter. The simulation protocols were then run thereafter.

All team pairings were randomised via drawing of poker cards—EMTs drawing the same number card would be paired together. Each team underwent 4 simulation runs, alternating between using the LUCAS-2 device (experimental group) and without the device (control group).

### Data collection and measurement

**Figure 2:**
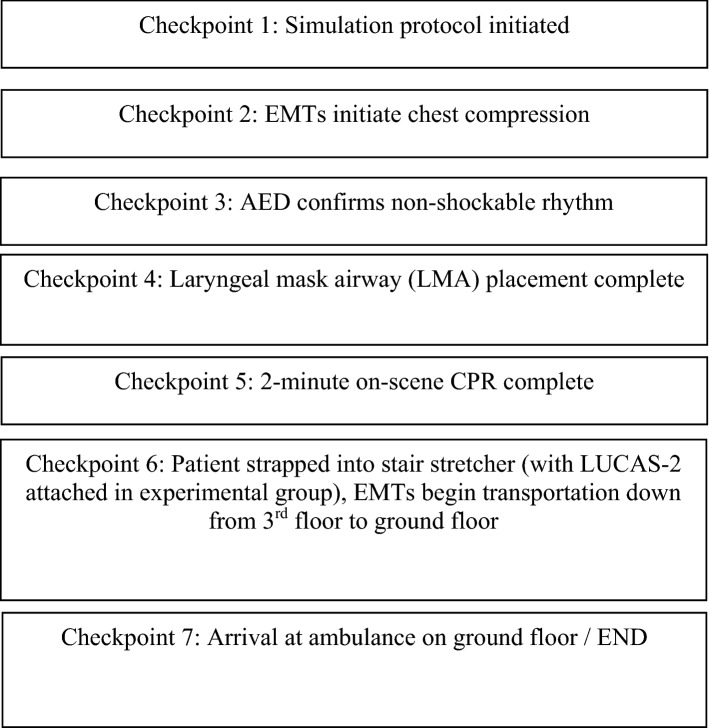
Different checkpoints of simulation runs.

The respective times taken for each phase of the simulation protocol were recorded (Fig. 2). CPR quality was separately assessed via the CPRmeter, and included the following variables: chest compression depth, rate, percentage of full recoil at end of compression, and no-flow time. The no-flow time was subsequently used to calculate the chest compression fraction via the following formula:

$$ {\text{Chest compression fraction}} = \left( {{\text{total resuscitation time}} - {\text{no-}}{\text{flow time}}} \right)/\left( {\text{total resuscitation time}} \right) $$

### Sample size estimation

Taking a significance level of 5% and power of 80%, we estimated a minimum sample size of 20 patients per group, for a total of 40 simulation runs.

### Statistical analysis

Data was analysed using SPSS v24.0 for Windows (SPSS; Chicago, IL, USA). Categorical and continuous variables were presented as frequencies with their corresponding percentages and medians with interquartile ranges respectively. Fisher’s exact tests were used to analyse the former and median test to analyse the latter. Statistical significance was taken at p < 0.05.

### Ethical approval

The Chang Gung Memorial Hospital Institutional Review Board approved this study (IRB No. 20170044B0). The research was performed in accordance with relevant guidelines/regulations in the manuscript. All procedures performed in studies involving human participants were in accordance with the ethical standards of the Chang Gung Medical Foundation Institutional Review Board and with the 1964 Helsinki declaration and its later amendments or comparable ethical standards.

### Informed consent

Informed consent was obtained from all participants.

## Results

A total of 40 simulations were run. Volunteer EMTs in both the LUCAS-2 and control groups did not vary significantly in terms of demographics. The LUCAS-2 group yielded a significantly higher chest compression fraction of 0.76 (p < 0.0001), though it required a longer time (~ 35 s) to prepare the patient prior to transport (p < 0.0001) and arrival at ambulance (p < 0.0001) compared to the control group. The CPR quality in terms of depth and rate did not differ significantly between the LUCAS-2 group and control group when taking into account the overall resuscitation period, though the CPRmeter recorded a reduction in proportion of compressions with adequate depth and rate (Table 2).

**Table 2:** Comparison between LUCAS-2 group and control group.

	Control	LUCAS-2	p value
**Volunteer demographics**			
Male (%)	90.0		NA
Working experience (years)	3.9		NA
**Elapsed time intervals from start of simulation (s)**			
Time to start of chest compression	26.5 (25.0, 28.5)	26.0 (24.0, 28.0)	0.2971
AED confirmation of non-shock	60.0 (58.5, 66.0)	59.5 (55.0, 64.0)	0.3467
LMA placement	73.5 (71.0, 76.0)	74.0 (65.5, 78.5)	0.4678
Completion time for 2-min CPR at scene	180.5 (179.0, 186.0)	180.0 (176.0, 184.5)	0.3482
Start of transportation^a^	203.0 (199.0, 208.0)	237.5 (231.0, 239.0)	< 0.0001
Arrival at ambulance^a^	259.0 (256.5, 265.5)	290.0 (283.5, 299.0)	< 0.0001
**Time taken to prepare for transport**^a^	21.0 (19.5, 22.0)	56.0 (51.0, 61.5)	< 0.0001
**Time taken for transportation**	57.0 (51.5, 60.5)	53.5 (52.0, 59.5)	0.3782
**CPR quality for overall resuscitation period**			
Total number of compressions throughout simulation, median (IQR)^a^	218.0 (206.5, 229.0)	312.5 (301.0, 321.0)	< 0.0001
Mean compression depth (mm)	57.5 (55.0, 61.0)	58.6 (54.8, 60.0)	0.9888
Proportion of compressions with adequate depth (%)	99.0 (93.0, 100.0)	92.5 (89.4, 94.0)	0.6892
Mean compression rate (min^−1^)	108.5 (102.5, 114.5)	106.3 (104.1, 109.3)	0.3121
Proportion of compressions with adequate rate (%)	95.5 (90.0, 100.0)	92.6 (87.7, 93.5)	0.9617
Proportion of compressions with full recoil (%)	94.5 (90.5, 99.0)	95.0 (90.9, 97.6)	0.3787
Chest compression fraction^a,b^	0.63 (0.62, 0.66)	0.76 (0.75, 0.78)	< 0.0001
**CPR quality at scene**			
Total number of compressions, median (IQR)	218.0 (206.5, 229.0)	217.0 (209.0, 224.0)	0.6757
Mean compression depth (mm)	57.5 (55.0, 61.0)	58.5 (53.0, 61.0)	1
Proportion of compressions with adequate depth (%)	99.0 (93.0, 100.0)	100.0 (94.0, 100.0)	0.2802
Mean compression rate (min^−1^)	108.5 (102.5, 114.5)	108.0 (104.0, 110.5)	0.6437
Proportion of compressions with adequate rate (%)	95.5 (90.0, 100.0)	99.5 (93.5, 100.0)	0.8506
Proportion of compressions with full recoil (%)	94.5 (90.5, 99.0)	94.5 (88.5, 97.0)	0.7907
**CPR quality during transportation**			
Total number of compressions, median (IQR)	0	93.5 (89.0, 102.5)	
Mean compression depth (mm)	0	57.0 (56.0, 59.5)	
Proportion of compressions with adequate depth (%)	0	79.0 (75.5, 83.5)	
Mean compression rate (min^−1^)	0	101.0 (101.0, 101.0)	
Proportion of compressions with adequate rate (%)	0	75.5 (73.5, 79.5)	
Proportion of compressions with full recoil (%)	0	97.0 (96.5, 99.0)	

*AED* automated external defibrillator, *LMA* laryngeal mask airway, *CPR* cardiopulmonary resuscitation.

^a^Statistical significance.

^b^Chest compression fraction = (total resuscitation time − no-flow time)/total resuscitation time. As such, chest compression fraction for control group = [period of CPR at scene (approx. “time to completion for 2-min CPR at scene” −“time to start of chest compression”)/total duration (“time to arrival at ambulance)]. Chest compression fraction for LUCAS group = {[period of CPR at scene (approx. “time to completion for 2-min CPR at scene” −“time to start of chest compression”) + “time taken for transportation”]/total duration (“time to arrival at ambulance)}.

When comparing quality of compressions performed by the LUCAS-2 device during transportation versus those done by our EMTs at scene, both had similar mean compression depths (p = 0.9629). The automated device yielded a more optimal proportion of compressions with full recoil (p = 0.0147) with comparable mean compression rate (101 min^− 1^ vs 108 min^−1^), though it fared worse in terms of proportion of compressions with adequate depth (p < 0.0001) and adequate rate (p = 0.0258).

The LUCAS-2 piston and the CPRmeter accelerometer at the end of the simulations remained in place.

## Discussion

The LUCAS-2 mechanical chest compression device promises to be an invaluable addition to the resuscitative efforts of emergency response teams worldwide, by providing standardised, automated compressions at the optimal depth and rate. In its current form, however, the usage of this device is limited to patients being transported in the supine position. This precludes patients who are transported down stairwells and across tight spaces from benefitting from this innovation. Our present study demonstrates that with a few small low-cost adaptations to the device, it can be applied during transportation down stairwells and across tight spaces.

Effective CPR requires uninterrupted chest compressions of adequate rate (100–120 min^−1^) and depth (5–6 cm). Our findings show that the LUCAS-2 device produces compressions of the appropriate rate (101 min^−1^) and depth (57.0 mm) throughout its usage during the simulation runs, and is not inferior to manual compressions by our highly-trained EMT teams. The proportion of recorded automated compressions with adequate depth and rate dipped to 79.0% and 75.5% respectively during the transportation phase; nevertheless when taking into account the entire resuscitation period, there were no significant differences in mean compression depth (p = 0.9888), proportion of compressions with adequate depth (p = 0.6892), mean compression rate (p = 0.3121), proportion of compressions with adequate rate (p = 0.9617), and proportion of compressions with full recoil (p = 0.3787) for both groups.

On further examination of CPR quality afforded by the LUCAS-2 during transportation, the device was found to be capable of producing optimal compressions of 57 mm depth at a rate of 101 min^−1^ during transport, 97% of which had full recoil. The proportion of compressions with adequate depth and rate however declined to 79.0% and 75.5% respectively—this could be due to occasional slippage of the piston against the CPRmeter accelerometer during the transport down the stairwell, leading to compressions which are not fully in the vertical plane. Such slippage would invariably occur due to some degree of sideways and rotational motion during non-supine transport, adversely affecting the quality of compressions which can be delivered by the device. Nevertheless, its use yielded a superior chest compression fraction of 0.76 as compared with 0.63 by the control group (p < 0.0001) for the entire resuscitation period. Even with suboptimal CPR quality during transport, the use of LUCAS-2 may still potentially improve post-OHCA survival rates given that some CPR is better than no CPR^24^—this would of course require confirmation in future clinical trials with real-world patients.

The application of the LUCAS-2 device to the manikin in our study costs an additional 35 s to the time taken to prepare for transport. Since time is muscle, especially in OHCAs, first responders should do a quick cost–benefit analysis to decide whether to use this automated device at all. The time taken for transportation in our study lasted almost 60 s for both groups, with the LUCAS-2 group yielding a better chest compression fraction despite the initial delay. Therefore, it suggests that in situations where EMTs can expect to require more than a minute for patient transportation from scene to ambulance, the benefits of continuous automated compressions during transport may outweigh the cost of an extra 35 s spent applying the device.

The chest compression fraction, defined as the proportion of resuscitation time without spontaneous circulation during which chest compressions were administered, has been shown to be an independent predictor of patient survival post-OHCA. For each 10% increment in chest compression fraction, the odds ratio of surviving to hospital discharge was 1.11^25^. Our findings thus suggest that the usage of the LUCAS-2 device in OHCA patients could potentially translate to higher survival rates. This is especially so for patients who require longer transportation times, due to their large body habitus or location in a place difficult to access. Furthermore, reduction of device deployment time may be improved with extensive application and further training in our novel method. The addition of a third person to the response team, as is the standard OHCA response team size, may also contribute to further cutting down on these 35 s.

The limitations of this study are that it has a relatively small sample size owing to its proof-of-concept nature. Future observational studies are required to confirm our findings’ applicability to real-life patients. Furthermore, design experts may be consulted to discuss how to optimise our adaptations to fit different patient body sizes. Last but not least, subsequent studies can be done to assess the efficacy of CPR in non-supine positions with respect to cerebral blood oxygenation via near-infrared spectroscopy^26^.

## Conclusion

Our novel adaptations to the LUCAS-2 device allow for uninterrupted compressions in patients being transported down stairwells, thus yielding better chest compression fractions for the overall resuscitation period. Whether potentially improved post-OHCA survival rates may be achieved requires confirmation in a real-world scenario study.

## Supplementary Information

Supplementary Video 1.

Supplementary Information 1.

## Data Availability

The datasets generated during and/or analysed during the current study are available from the corresponding author on reasonable request.
