# Supplementary material for: Shoulder strap fixation of LUCAS-2 to facilitate continuous CPR during non-supine (stair) stretcher transport of OHCAs patients
Source: Sci Rep. 2021 May 10;11:9858. doi: 10.1038/s41598-021-89291-4 (PMC8110788; doi:10.1038/s41598-021-89291-4)
Supplement: Supplementary file 2 — Supplementary Information 1. [file 41598_2021_89291_MOESM2_ESM.docx]

**Supplementary Video 1:** A sample video of the trial that shows novel adaptations of

LUCAS-2 mechanical chest compression device effectively maintain high quality CPR in

non-supine position for transportation down stairwells or in tight spaces.
